# Role of Single Nucleotide Polymorphisms of *KIF1B* Gene in HBV-Associated Viral Hepatitis

**DOI:** 10.1371/journal.pone.0045128

**Published:** 2012-09-18

**Authors:** Ahmed Al-Qahtani, Mashael Al-Anazi, Nisha A. Viswan, Nisreen Khalaf, Ayman A. Abdo, Faisal M. Sanai, Hamad Al-Ashgar, Mohammed Al-Ahdal

**Affiliations:** 1 Department of Infection and Immunity, Research Center, King Faisal Specialist Hospital & Research Center, Riyadh, Saudi Arabia; 2 Liver Disease Research Center, King Saud University, Riyadh, Saudi Arabia; 3 Department of Medicine, College of Medicine, King Saud University, Riyadh, Saudi Arabia; 4 Department of Hepatobiliary Science and Liver Transplantation, King Abdulaziz Medical City, Riyadh, Saudi Arabia; 5 Department of Medicine, King Faisal Specialist Hospital & Research Center, Riyadh, Saudi Arabia; 6 Department of Pathology and Laboratory Medicine, King Faisal Specialist Hospital & Research Center, Riyadh, Saudi Arabia; Drexel University College of Medicine, United States of America

## Abstract

**Background/Aim:**

Kinesin family member 1B (KIF1B) gene resides in the chromosomal region 1p36.22 and has been reported to have frequent deletions in a variety of human cancers. A recent genome wide association study (GWAS) study conducted on a Chinese population has reported the involvement of a KIF1B genetic variant in Hepatitis B virus (HBV)-related hepatocellular carcinoma (HCC). This study aims to investigate the significance of KIF1B genetic variations in HBV-associated hepatitis in patients of Saudi Arabian ethnicity.

**Methods:**

TaqMan genotyping assay was used to investigate the association of three SNPs (rs17401966, rs12734551, and rs3748578) in 584 normal healthy controls and 660 HBV-infected patients. The patients were categorized into inactive carriers (Case I), active carriers (Case II), Cirrhosis (Case III) and Cirrhosis-HCC (Case IV) sub-groups.

**Results:**

Since SNPs rs12734551 and rs3748578 are in strong linkage disequilibrium (LD) with rs17401966, only results for the latter SNP are reported. Therefore, the allele frequency of rs17401966 among HBV-infected patients and healthy controls were comparable and therefore, no significant association was observed (*P* = 0.2811, Odds Ratio (OR) 0.897). A similar analysis was performed among the different sub-groups in order to determine whether KIF1B SNPs were associated with the advancement of the disease. No significant differences were observed in any of the comparisons performed.

**Conclusion:**

Polymorphisms at KIF1B gene locus investigated in this study showed no significant association with HBV infection or with HBV-associated diseases such as liver cirrhosis or HCC.

## Introduction

Hepatitis B virus (HBV) infection constitutes a serious public health problem worldwide. Hundreds of millions of people are infected in almost all parts of the world and 1 million people die from HBV-associated diseases [1]. The epidemiology of HBV-associated hepatitis reflects a complex network of inter-related factors: host, viruses, and environment. As a result, the exact mechanisms that determine the disease outcome are yet to be clearly defined. Differences in susceptibility or resistance to HBV are attributed, at least in part, to variations in the human genome. Numerous genetic polymorphisms in different genes of various functions and regulatory sequences have been shown to be correlated with different manifestations of the disease. Among others, polymorphisms in different cytokine genes such as interleukin-1B (IL-1B), IL-16, interferon-γ (IFN-γ), tumor necrosis factor-α (TNF-α), and IL-10 have been shown to be linked to either susceptibility or resistance to HBV infection [2]–[6]. Other non-cytokine genes such as human leukocyte antigen (HLA), osteopontin, FAS, Estrogen receptor alpha, cytochrome P450, estrogen receptor, vitamin D3 receptor and micro RNAs were also demonstrated to be linked to HBV-related hepatitis [7]–[10].

**Table 1:** SNP marker information for KIF1B gene for all subjects studied (patients and healthy controls).

Gene	Chr. No.	SNPs	Position	ObsHET	PredHET	HWpval	MAF	Alleles
		rs17401966	10385471	0.314	0.318	0.6947	0.199	A:G
KIF1B	1	rs12734551	10391536	0.319	0.324	0.6736	0.203	T:G
		rs3748578	10420918	0.350	0.354	0.7405	0.229	G:A

Chr. No.: Chromosome Number; SNPs: Single Nucleotide Polymorphisms; Position: Chromosomal Position; ObsHET: Observed Heterozygosity; PredHET: Predicted Heterozygosity; HWpval: Hardy-Weinberg P-value; MAF: Minor Allele Frequency.

**Table 2:** Genotypic distribution for KIF1B gene polymorphisms when patient (Cases I+II+III+IV) group was compared to control group.

Gene	Chr. No.	SNPs	Genotype/Allele distribution	Controlsn = 584	Patientsn = 660	OR (95% C.I.)	?^2^	P-value
						0.897 (0.736–1.093)	1.16	0.281
			GG	25 (4.3%)	27 (4.1%)			
KIF1B	1	rs17401966	AG	192 (32.9%)	194 (29.7%)			
			AA	367 (62.8%)	432 (66.2%)			
			**G**	242 (20.7%)	248 (19.0%)			
			A	926 (79.3%)	1058 (81.0%)			
			GG+AG vs. AA			0.865 (0.685–1.093)	1.48	0.224

Risk allele marked in **BOLD** letter.

Recently, a new genetic locus was reported to be linked to HBV-associated hepatocellular carcinoma (HCC), using genome wide association study (GWAS) [11]. This locus (rs17401966) is a single nucleotide polymorphism (SNP), mapped to chromosome 1p36.22 and is in one of the intronic regions of the kinesin family member 1B (KIF1B) gene. Two adjacent SNPs (rs12734551 and rs3748578) showed evidence, albeit less strong, of association with HCC risk in HBV-infected patients and both SNPs were in strong linkage disequilibrium (LD) with rs17401966 [11]. This finding was consistent with cytogenetic studies that suggested a strong link between this chromosomal region, 1p36.22, and several kinds of cancer [12] including basal cell carcinoma [13], due to its frequent chromosomal loss. Moreover, a study conducted by Gasnereau and colleagues has reported that over-expression of miototic kinesin-like protein 2 (MKIp2), a member of the kinesin family, results in hepatocyte proliferation and thus contributes to tumor aggressiveness in HCC, thereby, making members of the kinesin family suitable candidates for genetic studies in order to determine their role in liver-related diseases [14]. KIF1B gene belongs to the kinesin superfamily of intermediate filaments involved in cytoskeletal structures of cells that are responsible for intracellular vesicular transport. Although several reports have shown that mutations in this gene were linked to several types of cancer, the underlying mechanism through which this gene contributes to carcinogenesis is still unclear. In addition, there is no information on the role of its polymorphisms on HBV infection within the Saudi population. The present study aimed at investigating the prevalence of genetic polymorphisms within the KIF1B gene in Saudi Arabian patients infected with HBV.

## Subjects and Methods

### Subjects

This study included 660 Saudi patients infected with HBV, who were recruited from three centers in Saudi Arabia (King Faisal Specialist Hospital and Research Center, Riyadh; Riyadh Military Hospital and King Khalid University Hospital, Riyadh) from August 2007 to August 2010. The study protocol was approved by the institutional review boards of all centers and conformed to the 1975 Declaration of Helsinki. All patients signed an informed consent prior to enrollment in the study, and their basic demographic data were recorded. Chronic HBV infection was diagnosed by the repeated detection of HBsAg over a period of 6 months. Patients were grouped into four different categories based on disease severity as follows: Case I - Inactive HBV carriers (patients who were positive for HBsAg and negative for HBeAg, with a persistently normal serum ALT levels); Case II - Active HBV carriers (patients who were positive for HBsAg, with elevated serum ALT levels); Case III - Patients with HBV infection and cirrhosis (cirrhosis was confirmed either by liver biopsy, clinical, biochemical or radiological evidence of cirrhosis) and Case IV - Patients with HBV infection and HCC with underlying cirrhosis. Diagnosis of HCC was made by computed tomography and/or magnetic resonance imaging of the liver, according to the published guidelines for the diagnosis and management of HCC [15]. A total of 584 subjects were randomly selected to constitute the uninfected healthy control group characterized by the absence of any known serological marker of HBV (HBsAg negative, anti HBe negative, and anti HBc negative) or the presence of anti-HBs antibodies in blood.

**Table 3:** Genotypic distribution for KIF1B gene polymorphisms when cases II+III+IV were compared to case I (inactive group).

Gene	Chr. No.	SNPs	Genotype/Allele distribution	Inactive n = 403	Active+ Cirrhosis+Cirrhosis-HCCn = 255	OR (95% C.I.)	?^2^	P-value
						0.917 (0.689–1.219)	0.36	0.549
			GG	15 (3.8%)	12 (4.7%)			
			AG	125 (31.6%)	69 (27.1%)			
KIF1B	1	rs17401966	AA	256 (64.6%)	174 (68.2%)			
			**G**	155 (19.6%)	93 (18.2%)			
			A	637 (80.4%)	417 (81.8%)			
			GG+AG vs. AA			0.851 (0.609–1.189)	0.89	0.345

Risk allele marked in **BOLD** letter.

**Table 4:** Genotypic distribution for KIF1B gene polymorphisms when cases III+IV groups were compared to case II (active group).

Gene	Chr. No.	SNPs	Genotype/Allele distribution	Active n = 184	Cirrhosis+ Cirrhosis-HCC n = 73	OR (95% C.I.)	?^2^	P-value
						1.118 (0.683–1.828)	0.20	0.657
			GG	9 (4.9%)	3 (4.2%)			
			AG	47 (25.7%)	22 (30.6%)			
KIF1B	1	rs17401966	AA	127 (69.4%)	47 (65.3%)			
			**G**	65 (17.8%)	28 (19.4%)			
			A	301 (82.2%)	116 (80.6%)			
			GG+AG vs. AA			1.206 (0.677–2.150)	0.40	0.525

Risk allele marked in **BOLD** letter.

### Genotyping of KIF1B SNPs

Genomic DNA was isolated from peripheral blood mononuclear cells using Gentra Pure Gene kit (Qiagen, Hilden, Germany) according to the manufacturer’s protocol. Patient and control samples were genotyped for the three polymorphic sites using 7900 HT Fast Real Time PCR System (Applied Biosystems, Foster City, CA, USA). All reagents required for the TaqMan assay including universal master mix, amplifying primers and probes were obtained from Applied Biosystems (Foster City, CA, USA). One allelic probe was labeled with FAM dye and the other with the fluorescent VIC dye. PCR was run in the TaqMan universal master mix at a probe concentration of 20x. The reaction was performed in a 96-well format in a total reaction volume of 25 µL using 20 ng of genomic DNA. The reaction plates were heated for 2 mins at 50°C and for 10 mins at 95°C, followed by 40 cycles of 95°C for 15 s and 60°C for 1.5 mins. The fluorescence intensity of each well in the TaqMan assay plate was subsequently read and fluorescence data files from each plate were analyzed by automated software (SDS 2.4).

### Statistical Analysis

All statistical analyses were performed using SPSS version 17.0 (SPSS Inc., Chicago, IL, USA). A χ^2^-test was used to compare the distribution of genotypes among patients and controls. The association between the KIF1B SNPs and the disease status were expressed in odds ratio (OR) and their 95% confidence intervals (CI). A p≤0.05 was considered to be statistically significant. The SNPs were tested for Hardy–Weinberg equilibrium (HWE) using the DeFinetti program (http://ihg.gsf.de/cgi-bin/hw/hwa1.pl). A cut-off p-value of 0.05 was set for HWE and SNPs were excluded from the study if minor allele frequency (MAF) <1%.

**Table 5:** Haplotypes of KIF1B gene variants when patient (Cases I+II+III+IV) group was compared to control group.

Gene	Block			Freq.	Case, ControlRatio Counts	Case, ControlFrequencies	Chi Square	P Value
KIF1B	rs17401966	rs12734551	rs3748578					
	A	T	G	0.764	1011.5: 308.5, 892.7: 279.3	0.766, 0.762	0.074	0.786
	G	G	A	0.192	240.6: 1079.4, 237.4: 934.6	0.182, 0.203	1.65	0.199
	A	T	A	0.033	52.2: 1267.8, 29.0: 1143.0	0.040, 0.025	4.289	0.038^♦^

♦This value is insignificant when Bonefforoni correction was applied.

## Results

TaqMan assays were performed on samples from 403 Inactive HBV carriers (Case I), 184 Active HBV carriers (Case II), 55 cirrhotic patients (Case III), 18 cirrhosis-HCC patients (Case IV) and 584 control subjects.

**Table 6:** Haplotypes of KIF1B gene variants when cases II+III+IV groups were compared to case I (inactive group).

Gene	Block			Freq.	Case, ControlRatio Counts	Case, ControlFrequencies	Chi Square	P Value
KIF1B	rs17401966	rs12734551	rs3748578					
	A	T	G	0.764	397.6: 118.4, 610.8: 193.2	0.770, 0.760	0.205	0.651
	G	G	A	0.182	91.8: 424.2, 148.7: 655.3	0.178, 0.185	0.103	0.748
	A	T	A	0.04	20.3: 495.7, 33.1: 770.9	0.039, 0.041	0.027	0.870

**Table 7:** Haplotypes of KIF1B gene variants when Cases III+IV groups were compared to case II (active group).

Gene	Block			Freq.	Case, ControlRatio Counts	Case, ControlFrequencies	Chi Square	P Value
KIF1B	rs17401966	rs12734551	rs3748578					
	A	T	G	0.772	109.8: 36.2, 288.8: 81.2	0.752, 0.781	0.49	0.484
	G	G	A	0.18	28.0: 118.0, 64.9: 305.1	0.192, 0.175	0.184	0.668
	A	T	A	0.037	6.2: 139.8, 13.0: 357.0	0.043, 0.035	0.157	0.692

The genotypic distribution of KIF1B SNPs (rs17401966, rs12734551 and rs3748578), were found to be in Hardy-Weinberg equilibrium (HWE) with a MAF>1% within the Saudi population (Table 1). Genotyping was performed for the three SNPs on all samples tested in this study, however, since SNPs rs12734551 and rs3748578 are in strong linkage disequilibrium with SNP rs17401966 [11], only data related to SNP rs17401966 was presented in this report.

The distribution of genotypes among patients suffering from HBV infection and controls were found to be comparable for rs17401966 (OR = 0.897; 95% CI, 0.736–1.093) (Table 2) and no significant association was observed in relation to the disease.

A similar analysis was done by comparing Case II+III+IV to the inactive carrier group (Case I) and also comparing Case III+IV to active carriers (Case II), in order to determine if rs17401966 was associated with progression of HBV infection to cirrhosis and HCC, however, no significant association was observed in any of these comparisons (Tables 3 and 4).

Haplotype analysis was done among HBV infected patients versus control group, using Haploview 4.2 software, and three haplotypes were observed (Table 5). Out of which, the least frequent haplotype, ATA (rs17401966, rs12734551 and rs3748578) was found to be significant with a frequency of 0.033 (χ^2^ = 4.289; p = 0.038). However, when the Bonefforoni correction method was applied, the significant association was lost.

Furthermore, when such analysis was done amongst Case II+III+IV compared to the inactive carrier group (Case I) and amongst Case III+IV compared to Active carriers (Case II), three haplotypes were observed for each comparisons. However, none of the haplotypes were found to be significant and their frequency of occurrence, χ^2^ and p-value are presented in Tables 6 and 7.

## Discussion

The key step in carrying out a candidate gene study is the identification of a suitable candidate gene that may plausibly be involved in the process or disease under study. The candidate gene approach for discovering genetic markers uses experimentally derived a priori knowledge about the disease, and in this study KIF1B gene and its polymorphisms were selected for association study with HBV infection and its progression to HCC in Saudi Arabian patients.

KIF1B gene belongs to the kinesin superfamily that encodes proteins responsible for the intracellular transport of mitochondria and synaptic vesicular precursors [16]. Mutations within this gene have been reported in many neuropathological conditions such as Charcot-Marie-Teeth (CMT) type 2A peripheral neuropathy [17], multiple sclerosis [18] and predisposition to many neural and non-neural tumors [19]. In addition, KIF1B has been reported to have a haploinsufficient tumor-suppressor function in primary neuroblastomas and pheochromocytomas by acting downstream from EglN3 prolyl hydroxylase [20], [21].

The present case-control study investigates the plausible role of three single nucleotide polymorphisms (rs17401966, rs12734551 and rs3748578) in KIF1B gene and its association with HBV infection, its chronicity or its progression to HCC. However, none of the three SNPs in question were found to have any association neither to susceptibility to HBV infection nor to its progression to cirrhosis or HCC. This finding contradicts with a recent GWAS study conducted by Zhang et al., 2010 in subjects from five different regions of China, which has suggested that KIF1B rs17401966 confers susceptibility to HBV-induced HCC [11]. In the GWAS study, haplotype GGA (rs17401966, rs12734551 and rs3748578) was found to be associated with HCC risk (p - 6.0×10^−6^). While, in the current study, none of the haplotypes showed any evidence of association with HCC risk, except for haplotype ATA, which was found to have a significant association with respect to HBV infection with a p-value of 0.038. However, after applying Bonferroni correction while testing 3 haplotypes, a p value <0.017 is needed for a statistical significance to be accepted, which was not reached in the present study. Interestingly, another Chinese study showed results that were in agreement with the present study, and concluded that neither SNP rs17401966 nor any haplotypes studied were involved in progression to chronic hepatitis B [22].

When the allele frequency of the SNP rs17401966 in this study (A = 0.8, G = 0.2), was compared with the frequencies of 11 populations found in the NCBI HapMap Database, a comparable allele frequency was observed amongst the Asian and the Mexican population, but was slightly lower than the Yoruban population.

Recent studies have reported the possible role of members from the kinesin family, such as kinesin-1, to be involved in promoting virus infection by aiding in the uncoating of the virus, disrupting the viral capsid and dislocating nucleoporins. Thus, this process is hypothesized to increase the nuclear envelope permeability, and thereby allowing entry of viral DNA into the nucleus [23]. In addition, a study conducted by Danquah and colleagues suggested that kinesin plays an important role in the trafficking of the virus in infected insect cells [24]. However, the current study fails to identify any significant association between KIF1B gene and HBV infection, a finding at odds to the recently published GWAS study performed on a Chinese ethnic population [11]. This could indicate that the role of KIF1B, if any, in the development of HBV-associated HCC is population dependent as the current study was done on Saudi Arabian subjects. Also, other reasons that could account for the difference in association of rs17401966 amongst the Chinese and Saudi populations could be virus-related factors. Such factors include HBV genotype since the predominant genotype in Saudi Arabia is genotype D [25] whereas genotype C predominates in China [26], viral load and divergence in nucleotide sequence. Moreover, other reported genetic markers that contribute to disease outcome in chronic HBV infected patients may vary between both populations. Examples of such markers include the leukocyte antigen HLA gene variants [27], [28], the TNF-α promoter polymorphisms [29] and RANTES gene polymorphisms [30].

A major concern in interpreting results presented in this study is the effect of population stratification as the frequency of an unmeasured risk factor for disease differs by ethnicity [31]. However, population stratification is not a serious threat to the reliability of cohort and case-control studies, at least in certain ethnic populations such as non-Hispanics of European descent [32]. Confounding by population stratification may not be a major concern in the Saudi patients included in this study due to the fact that our study is a controlled one. Replication in a different population is one of the solutions if population stratification does indeed cause serious bias [33]. The similarity of our findings to those shown by the Chinese study [22], which included patients with a completely different ethnicity supports ours view and fulfills this replication rule. The second recommended solution to avoid the confounding effect of population stratification is a genomic control [31], which uses markers unrelated to disease to correct for the bias [34]. However, we believe that although this method provides an acceptable way to enhance the credibility of studies with unrelated controls by ruling out even the remote possibility of bias, it is particularly useful when ethnic differences are important and self-report is very difficult, which is not the case in Saudi patients studied in this report.

In conclusion, no significant association was observed between KIF1B genetic polymorphisms with either HBV infection or to its progression to HCC in Saudi Arabian population.

## References

[1] Fung J, Lai CL, Yuen MF (2010) Hepatitis B virus DNA and hepatitis B surface antigen levels in chronic hepatitis B. Expert Rev Anti Infect Ther. 8: 717–726. 10.1586/eri.10.45 20521898

[2] Migita K, Maeda Y, Abiru S, Nakamura M, Komori A, et al (2007) Polymorphisms of interleukin-1beta in Japanese patients with hepatitis B virus infection. J Hepatol 46: 381–386. 17126449 10.1016/j.jhep.2006.09.015

[3] Abbott W, Gane E, Winship I, Munn S, Tukuitonga C (2007) Polymorphism in intron 1 of the interferon-gamma gene influences both serum immunoglobulin E levels and the risk for chronic hepatitis B virus infection in Polynesians. Immunogenetics 59: 187–195. 17211638 10.1007/s00251-006-0184-4

[4] Suneetha PV, Sarin SK, Goyal A, Kumar GT, Shukla DK, et al (2006) Association between vitamin D receptor, CCR5, TNF-alpha and TNF-beta gene polymorphisms and HBV infection and severity of liver disease. J Hepatol 44: 856–863. 16545485 10.1016/j.jhep.2006.01.028

[5] Li S, Deng Y, Chen ZP, Huang S, Liao XC, et al (2011) Genetic polymorphism of interleukin-16 influences susceptibility to HBV-related hepatocellular carcinoma in a Chinese population. Infect Genet Evol 11: 2083–2088. 22019522 10.1016/j.meegid.2011.09.025

[6] Cheong JY, Cho SW, Hwang IL, Yoon SK, Lee JH, et al (2006) Association between chronic hepatitis B virus infection and interleukin-10, tumor necrosis factor-a gene promoter polymorphisms. J Gastroenterol Hepatol 21: 1163–1169. 16824070 10.1111/j.1440-1746.2006.04304.x

[7] Höhler T, Gerken G, Notghi A, Lubjuhn R, Taheri H, et al (1997) HLA-DRB1*1301 and *1302 protect against chronic hepatitis B. J Hepatol. 26: 503–507. 10.1016/s0168-8278(97)80414-x 9075656

[8] Xie H, Song J, Du R, Liu K, Wang J, et al (2007) Prognostic significance of osteopontin in hepatitis B virus-related hepatocellular carcinoma. Dig Liver Dis 39: 167–172. 17161983 10.1016/j.dld.2006.10.015

[9] Akkız H, Bayram S, Bekar A, Akgöllü E, Ulger Y (2011) A functional polymorphism in pre-microRNA-196a-2 contributes to the susceptibility of hepatocellular carcinoma in a Turkish population: a case-control study. J Viral Hepat 18: e399–407. 21692953 10.1111/j.1365-2893.2010.01414.x

[10] Yan Z, Tan W, Dan Y, Zhao W, Deng C, et al (2012) Estrogen receptor alpha gene polymorphisms and risk of HBV-related acute liver failure in the Chinese population. BMC Med Genet 13(1): 49. 22727021 10.1186/1471-2350-13-49 PMC3412699

[11] Zhang H, Zhai Y, Hu Z, Wu C, Qian J, et al (2010) Genome-wide association study identifies 1p36.22 as a new susceptibility locus for hepatocellular carcinoma in chronic hepatitis B virus carriers. Nat Genet. 42: 755–758. 10.1038/ng.638 20676096

[12] Bagchi A, Mills AA (2008) The quest for the 1p36 tumor suppressor. Cancer Res 68: 2551–2556. 18413720 10.1158/0008-5472.CAN-07-2095 PMC2980353

[13] Stacey SN, Gudbjartsson DF, Sulem P, Bergthorsson JT, Kumar R, et al (2008) Common variants on 1p36 and 1q42 are associated with cutaneous basal cell carcinoma but not with melanoma or pigmentation traits. Nat Genet 40: 1313–1318. 18849993 10.1038/ng.234

[14] Gasnereau I, Boissan M, Margall-Ducos G, Couchy G, Wendum D, et al (2012) KIF20A mRNA and its product MKlp2 are increased during hepatocyte proliferation and hepatocarcinogenesis. Am J Pathol 180: 131–140. 22056911 10.1016/j.ajpath.2011.09.040

[15] Abdo AA, Karim HA, Al Fuhaid T, Sanai FM, Kabbani M, et al (2006) Saudi Gastroenterology Association guidelines for the diagnosis and management of hepatocellular carcinoma: summary of recommendations. Ann Saudi Med 26: 261–265. 16880728 10.5144/0256-4947.2006.261 PMC6074501

[16] Nangaku M, Sato-Yoshitake R, Okada Y, Noda Y, Takemura R, et al (1994) KIF1B, a novel microtubule plus end-directed monomeric motor protein for transport of mitochondria. Cell 79: 1209–1220. 7528108 10.1016/0092-8674(94)90012-4

[17] Zhao C, Takita J, Tanaka Y, Setou M, Nakagawa T, et al (2001) Charcot-Marie-Tooth disease type 2A caused by mutation in a microtubule motor KIF1Bbeta. Cell 105: 587–597. 11389829 10.1016/s0092-8674(01)00363-4

[18] Aulchenko YS, Hoppenbrouwers IA, Ramagopalan SV, Broer L, Jafari N, et al (2008) Genetic variation in the KIF1B locus influences susceptibility to multiple sclerosis. Nat Genet 40: 1402–1403. 18997785 10.1038/ng.251

[19] Yeh IT, Lenci RE, Qin Y, Buddavarapu K, Ligon AH, et al (2008) A germline mutation of the KIF1B beta gene on 1p36 in a family with neural and nonneural tumors. Hum Genet 124: 279–285. 18726616 10.1007/s00439-008-0553-1

[20] Munirajan AK, Ando K, Mukai A, Takahashi M, Suenaga Y, et al (2008) KIF1Bbeta functions as a haploinsufficient tumor suppressor gene mapped to chromosome 1p36.2 by inducing apoptotic cell death. J Biol Chem 283: 24426–24434. 18614535 10.1074/jbc.M802316200 PMC3259808

[21] Schlisio S, Kenchappa RS, Vredeveld LC, George RE, Stewart R, et al (2008) The kinesin KIF1Bbeta acts downstream from EglN3 to induce apoptosis and is a potential 1p36 tumor suppressor. Genes Dev 22: 884–893. 18334619 10.1101/gad.1648608 PMC2279200

[22] Zhong R, Tian Y, Liu L, Qiu Q, Wang Y, et al (2012) HBV-related hepatocellular carcinoma susceptibility gene KIF1B is not associated with development of chronic hepatitis B. PLoS One. 7(2): e28839. 10.1371/journal.pone.0028839 PMC3283615 22363396

[23] Strunze S, Engelke MF, Wang IH, Puntener D, Boucke K, et al (2011) Kinesin-1-mediated capsid disassembly and disruption of the nuclear pore complex promote virus infection. Cell Host Microbe 10: 210–223. 21925109 10.1016/j.chom.2011.08.010

[24] Danquah JO, Botchway S, Jeshtadi A, King LA (2012) Direct interaction of baculovirus capsid proteins VP39 and EXON0 with kinesin-1 in insect cells determined by fluorescence resonance energy transfer-fluorescence lifetime imaging microscopy. J Virol 86: 844–853. 22072745 10.1128/JVI.06109-11 PMC3255796

[25] Abdo AA, Al-Jarallah BM, Sanai FM, Hersi AS, Al-Swat K, et al (2006) Hepatitis B genotypes: relation to clinical outcome in patients with chronic hepatitis B in Saudi Arabia. World J Gastroenterol 12(43): 7019–7024. 17109498 10.3748/wjg.v12.i43.7019 PMC4087347

[26] Norder H, Couroucé AM, Coursaget P, Echevarria JM, Lee SD, et al (2004) Genetic diversity of hepatitis B virus strains derived worldwide: genotypes, subgenotypes, and HBsAg subtypes. Intervirology 47(6): 289–309. 15564741 10.1159/000080872

[27] Guo X, Zhang Y, Li J, Ma J, Wei Z, et al (2011) Strong influence of human leukocyte antigen (HLA)-DP gene variants on development of persistent chronic hepatitis B virus carriers in the Han Chinese population. Hepatology 53(2): 422–428. 21274863 10.1002/hep.24048 PMC3056070

[28] Yan ZH, Fan Y, Wang XH, Mao Q, Deng GH, et al (2012) Relationship between HLA-DR gene polymorphisms and outcomes of hepatitis B viral infections: A meta-analysis. World J Gastroenterol 18(24): 3119–3128. 22791948 10.3748/wjg.v18.i24.3119 PMC3386326

[29] Qiu B, Wang X, Zhang P, Shi C, Zhang J, et al.. (2012) Association of TNF-α promoter polymorphisms with the outcome of persistent HBV infection in a northeast Chinese Han population. Acta Biochim Biophys Sin (Shanghai) [Epub ahead of print]) 10.1093/abbs/gms046 22695741

[30] Al-Qahtani A, Alarifi S, Al-Okail M, Hussain Z, Abdo A, et al (2012) RANTES gene polymorphisms (-403G>A and -28C>G) associated with hepatitis B virus infection in a Saudi population. Genet Mol Res 11(2): 855–862. 22576913 10.4238/2012.April.10.1

[31] Thomas DC, Witte JS (2002) Point: population stratification: a problem for case-control studies of candidate-gene associations? Cancer Epidemiol Biomarkers Prev 11(6): 505–512. 12050090

[32] Wacholder S, Rothman N, Caporaso N (2002) Counterpoint: bias from population stratification is not a major threat to the validity of conclusions from epidemiological studies of common polymorphisms and cancer. Cancer Epidemiol Biomarkers Prev 11(6): 513–520. 12050091

[33] Wacholder S, Rothman N, Caporaso N (2000) Population stratification in epidemiologic studies of common genetic variants and cancer: quantification of bias. J Natl Cancer Inst 92(14): 1151–1158. 10904088 10.1093/jnci/92.14.1151

[34] Satten GA, Flanders WD, Yang Q (2001) Accounting for unmeasured population substructure in case-control studies of genetic association using a novel latent-class model. Am J Hum Genet 68(2): 466–477. 11170894 10.1086/318195 PMC1235279

